# Effects of emergency cerclage on the neonatal outcomes of preterm twin pregnancies compared to preterm singleton pregnancies: A neonatal focus

**DOI:** 10.1371/journal.pone.0208136

**Published:** 2018-11-26

**Authors:** Sang Hoon Chun, Jiyoung Chun, Keun-Young Lee, Tae-Jung Sung

**Affiliations:** 1 Department of Pediatrics, Kangnam Sacred Heart Hospital, Hallym University Medical Center, Seoul, Korea; 2 Department of Obstetrics and Gynecology, Kangnam Sacred Heart Hospital, Hallym University Medical Center, Seoul, Korea; University of Liverpool, UNITED KINGDOM

## Abstract

**Objective:**

To evaluate the efficacy and safety of emergency cerclage (EC) in preterm twins by comparing neonatal outcomes of preterm twins with those of preterm singletons.

**Study design:**

This is a single-institution retrospective study of preterm infants born to women who underwent EC from 2008 to 2014. We compared various maternal and neonatal factors. The primary and secondary goals were to compare the maternal and neonatal morbidities and neonatal mortality, respectively.

**Results:**

One hundred fifty-three infants were included comprising 32(21%) twins and 121(79%) singletons. The mean gestational age (GA) at the time of EC and the number of days from EC to delivery were not significantly different (47.9±27.5 vs. 48.3±35.5). The rate of preterm delivery at ≤32 weeks GA (69% vs. 79%) and ≤28 weeks GA (50% vs. 55%), and other prematurity-associated morbidities were not significantly different. The survival rate during hospitalization was 75% (24/32) in twins and 88% (107/121) in singletons (*P* = 0.054). Death within 7 days after birth occurred in 8 twins (25%) and 7 singletons (6%) (*P* = 0.001). All of the infants were <1,000 g with a GA of ≤27 weeks.

**Conclusion:**

Compared to EC in singleton pregnancies, EC in twin pregnancies resulted in a higher mortality rate for preterm babies. EC might be considered a salvage procedure for selective twin pregnancies with cervical insufficiency.

## Introduction

The rate of twin births has increased in recent years to account for more than 3% of all live births, although it varies by maternal age and ethnicity [1, 2]. Twin pregnancies are a high-risk obstetric population, and the most common adverse outcomes in this population is related to preterm delivery. The risk of preterm birth in twins was reported to be 12 times higher than that in singleton births [1, 3]. Since preterm birth remains the leading cause of neonatal morbidities and mortality despite tremendous improvements in the field of neonatal care, every effort is needed to prevent preterm birth both in twins and singletons.

Cervical insufficiency, one of the pathological processes known to contribute to the presence of supraphysiological stimuli in the uterus in twin pregnancies, might further increase the risk of preterm birth [4]. The rate of preterm delivery with cervical insufficiency is known to be higher in twin pregnancies (5%) than in singleton pregnancies (0.05–1.8%) [5]. To reduce perinatal complications related to preterm delivery in twin pregnancies with cervical insufficiency, proper management of a short cervix should be considered to prolong these pregnancies.

Although variable treatment modalities including bed rest, limitation of home activities, prophylactic tocolysis, progesterone, or cervical cerclage have been suggested, these techniques have shown different results [6–8]. In addition, cervical cerclage in singleton pregnancies is currently widely accepted, but the use of this procedure in twin pregnancies is still debated [9, 10].

Emergency cerclage (EC) or rescue cerclage, which involve cerclage placement in women with a painless dilated cervix and amniotic membranes prolapsed into the vagina, is a challenging procedure to perform successfully [11]. Numerous studies have shown promising results regarding the benefits of EC in singleton infants [12–15]. However, few studies have been published on EC in multiple pregnancies [16–21]. In addition, due to the risk of cerclage-induced complications and preterm births, the dilemma of cerclage in mid-trimester twin pregnancies might be a concerning but very important issue for both obstetricians and neonatologists.

Hence, the aim of this study was to evaluate the effectiveness and safety of EC in twin pregnancies by comparing the neonatal morbidity and mortality in twins with those in singletons born prematurely at <37 weeks of gestational age (GA) to women with cervical insufficiency.

## Methods

A retrospective single-institution cohort study of infants whose mothers underwent EC at Hallym University Medical Center between January 2008 and December 2014 was conducted. Infants with congenital infections or fetal anomalies, twin pregnancies in which one fetus died before delivery, and twins with delayed internal delivery were excluded. For chorionicity of the twin pregnancies, we first attempted to find any description from the medical charts; however, due to the innate limitations of retrospective studies, we were only able to acquire descriptions of chorionicity for a small number of cases. We have thought about this issue thoroughly, and after concluding that the indications of the operation method did not change according to chorionicity, we decided not to add the chorionicity data.

Various maternal and neonatal factors were compared between twins and singletons by medical chart reviews. The study protocol was approved by the institutional review board of Hallym University before the investigation began. Informed consent was waived by the institutional review board due to the retrospective nature of this study.

Generally, cervical insufficiency was diagnosed as painless dilatation and/or effacement of the cervix in the absence of contractions or bleeding in the second trimester with a cervical length <25 mm measured by a transvaginal sonogram with prior second-trimester or early third-trimester fetal losses [22]. However, the more widely accepted definition is suggested by current ACOG guidelines, which indicate that cervical insufficiency should refer to a very specific group of women delivering in the early second trimester without contractions [23]. Although the diagnosis of cervical insufficiency in twin pregnancies was difficult, we tried to follow the same ACOG diagnostic criteria for singleton pregnancies, which were based on a history of painless cervical dilation after the first trimester without contractions or labor. Based on the EC criteria, EC was performed for mothers with a painless dilated cervix ≥1.0 cm and visible prolapsed amniotic membranes beyond the external cervical os confirmed by speculum examination in the second trimester, i.e., 13–25 weeks. When the cervix was ≥2 cm dilated on manual or speculum examination, routine amniocentesis to assess for subclinical intra-amniotic infection was performed. If intrauterine infection was confirmed, obstetricians did not perform cerclage as these pregnancies were at increased risk of preterm delivery and other pregnancy complications. Other contraindications for performing EC were uterine contractions, vaginal bleeding, and fetal anomalies. Emergency cervical cerclage was performed as follows. First, the bulging membrane was pushed back into the uterine cavity with a uni-concave inflated balloon. Then, the McDonald technique was performed with one suture using 5 mm Mersilene tape placed in a purse-string fashion; the balloon was then deflated and the purse-string suture was tied as the instrument was withdrawn [24].

External tocodynamometry was performed in all mothers to rule out preterm labor or impending miscarriage before cerclage placement. If prolapsed membranes beyond the external os were identified, then amnioreduction was performed during amniocentesis to reduce the tension on the membranes. Prophylactic tocolysis was performed preoperatively for most of the mother, and prophylactic broad-spectrum antibiotics were administered perioperatively for at least 5 consecutive days. Vaginal and/or endocervical swabs were obtained before EC to rule out infection, and when bacterial colonization was confirmed, targeted antibiotics were started for a certain period time.

Maternal factors including maternal age, nulliparity, previous preterm delivery, in vitro fertilization, mode of delivery, antenatal steroid use, chorioamnionitis, preterm premature rupture of membranes (PPROM) after cerclage, GA at cerclage placement, number of days from EC to delivery, vaginal progesterone use, and death within 7 days after birth were compared between twins and singleton infants. Neonatal variables including GA, birth weight, Apgar scores at 1 minute and 5 minutes, incidence of respiratory distress syndrome (RDS), bronchopulmonary dysplasia (BPD), patent ductus arteriosus (PDA), and early sepsis, survival rate, early death, (i.e., death within 7 days after delivery), and abnormal brain sonography findings were also assessed.

PPROM was defined as gross rupture of amniotic fluid, visualization of amniotic fluid on a sterile speculum examination, and a positive nitrazine test result. Chorioamnionitis was defined as follows: maternal fever ≥38°C plus one of the following: maternal tachycardia (>100 beats/min), fetal tachycardia (>160 beats/min), marked leukocytosis (>15,000 cells/mm^3^), or a foul odor of the amniotic fluid. Early sepsis was defined as culture-proven sepsis within the first 7 days of NICU hospitalization.

Neonatal mortality is known to be higher in preterm twins than in singletons [25] and is therefore a serious problem in the NICU. Hence, our second goal was to analyze neonatal mortality as well as early mortality (i.e., death within 7 days of the hospitalization stay following delivery) to evaluate the short-term effects of EC.

Statistical analyses were performed using SPSS (version 23.0, IBM, Armonk, NY, USA). The results are expressed either as numbers and percentages or as the mean ± standard deviation for normally distributed continuous variables. Student’s *t* test or the Mann-Whitney U-test was used to evaluate means, and the Chi-squared test was used to evaluate frequencies. Statistical significance was achieved when *P* values were <0.05.

## Results

**Fig 1:**
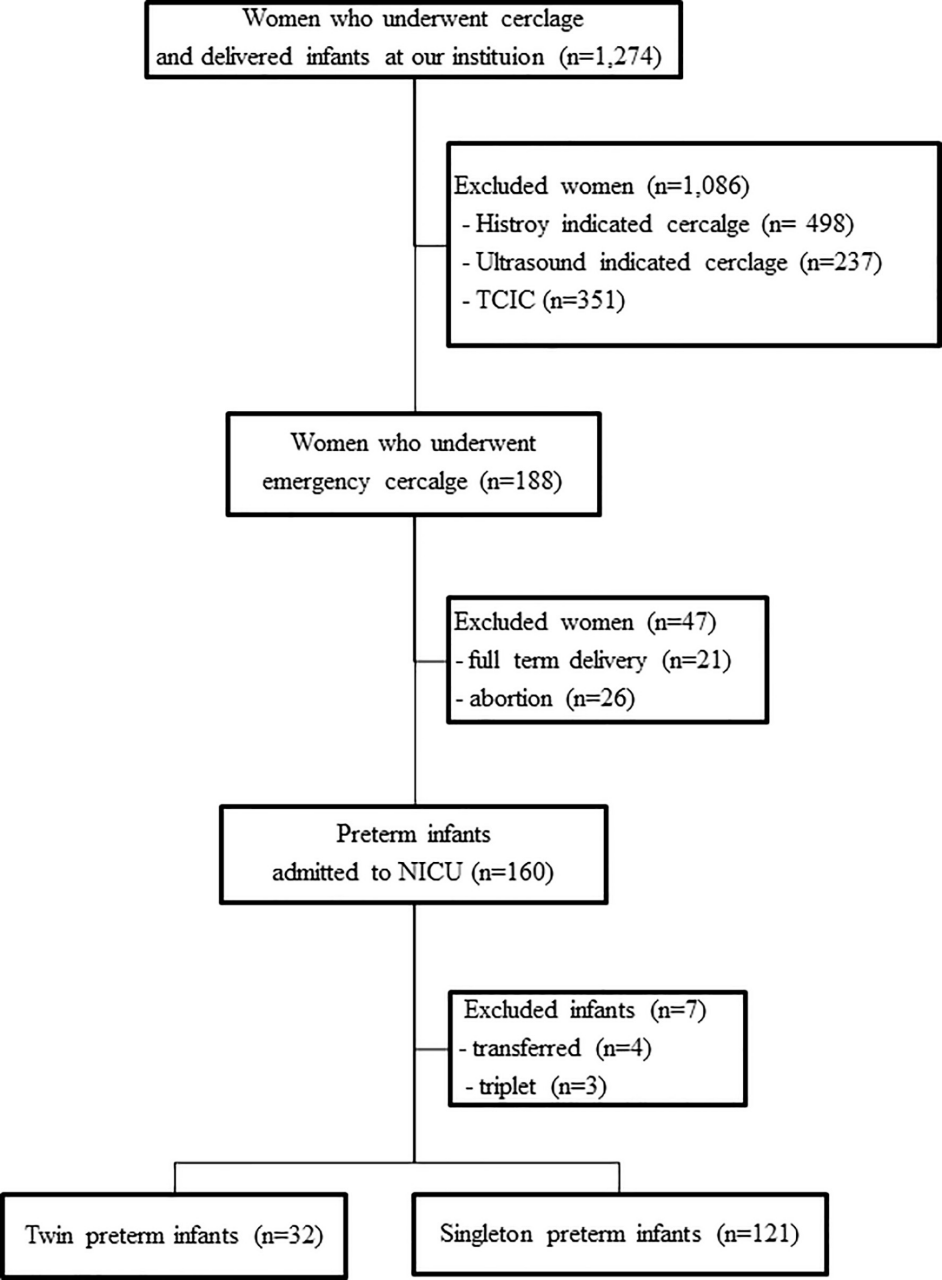
Schematic view of the study population. Abbreviations: TCIC, transabdominal cervico-isthmic cerclage; NICU, neonatal intensive care unit.

A total of 2,213 women with cervical insufficiency underwent cervical cerclage, and 1,274 women delivered infants at this institution. Among the women who underwent EC, 21 women delivered full term infants, with 19 singleton pregnancies and 2 twin pregnancies. Twenty-six women had fetal loss, including 22 singleton pregnancies and 4 twin pregnancies. As a result, 160 infants from 141 women were admitted to the NICU. A total of 160 preterm infants [34 twins (21%), 3 triplets (2%), 123 singletons (77%)] were delivered by women who underwent EC and were admitted to the neonatal intensive care unit (NICU) at the same hospital. Seven preterm infants were excluded from the final analysis: 2 who were transferred to other institutions for surgery, 2 due to the parents’ request, and 3 triplets (Fig 1).

**Fig 2:**
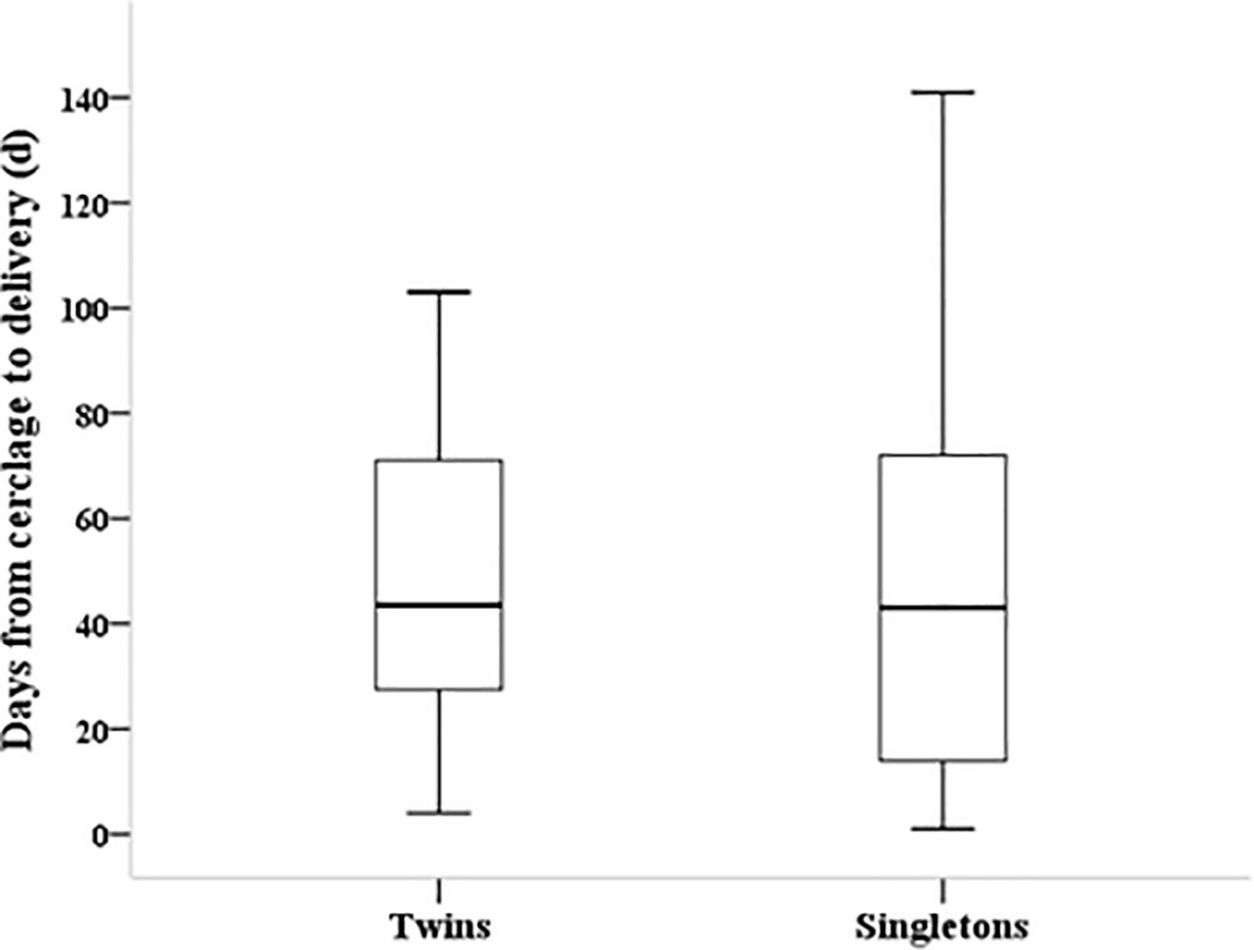
Comparison of days from cervical cerclage to delivery between the two groups.

The mean maternal age in this study was 32.6±3.1 among mothers of twins and 32.9±4.0 among mothers of singletons (*P* = 0.681). The incidence of nulliparity, in vitro fertilization, and Cesarean section were significantly higher in twin pregnancies than in singleton pregnancies (*P* <0.01 for all). GA at the time of cerclage was almost the same between the two groups, and the incidence of previous preterm delivery was not different between the two groups. Prophylactic tocolysis was performed on almost all of the mothers in the study population (100% in twin pregnancies vs. 96% in singleton pregnancies), and vaginal progesterone was administered to 20(53%) twin mothers and 71(59%) singleton mothers (*P* = 0.695) (Table 1). The number of days from cerclage to delivery was not significantly different between the two groups (47.9±27.5 days vs. 48.3±35.5days, *P* = 0.952) (Fig 2).

**Table 1:** Comparison of maternal clinical variables between the two groups.

	Twins (N = 32)	Singletons (N = 121)	*P* value
Maternal age*	32.6±3.1	32.9±4.0	0.681
Nulliparous (%)	16(50.0)	22(18.2)	<0.01
In vitro fertilization (%)	22(68.8)	14(11.7)	<0.01
Cesarean section (%)	30(93.8)	51(42.1)	<0.01
Antenatal steroids (%)	23(71.9)	96(79.3)	0.366
Chorioamnionitis (%)	18(56.3)	64(52.9)	0.735
PPROM after cerclage (%)	4(12.5)	12(9.9)	0.675
Gestational age at cerclage* (week)	22.3±1.8	21.8±3.0	0.327
Days from cerclage to delivery*(d)	47.9±27.5 (4–103)	48.3±35.5 (1–141)	0.952
Previous preterm delivery (%)	6(18.8)	28(23.1)	0.588
Vaginal progesterone (%)	20(62.5)	71(58.7)	0.695
Tocolysis (%)	32(100)	116(95.9)	0.242

*Values are mean±SD

Abbreviation: PPROM; preterm premature rupture of membranes

The mean GA of twins and singletons were 29.3±4.3 weeks and 28.6±3.8 weeks, respectively (*P* = 0.343). The incidence of preterm birth before 32 weeks (69% vs. 79%, *P* = 0.288) and the incidence of preterm birth before 28 weeks were not different between the two groups (50% vs. 55%, *P* = 0.867). Regarding the birth weight, there were no significant differences in the rate of very low birth weight infants (<1,500 g, VLBWI) (69% vs. 61%, *P* = 0.430) or extremely low birth weight infants (<1,000 g, ELBWI) (41% vs. 38%, *P* = 0.787) between the two groups. The incidences of prematurity-related morbidities, including RDS, BPD, PDA, and early sepsis, were not different between the two groups (Table 2). Abnormal brain sonography revealed intraventricular hemorrhage (≥ grade 2) ± periventricular leukomalacia, at similar rates between the two groups.

**Table 2:** Comparison of Neonatal variables between the two groups.

	Twins (N = 32)	Singletons (N = 121)	*P* value
Gestational age* (week)	29.3±4.3	28.6±3.8	0.343
≤28 weeks (%)	16(50.0)	66(54.5)	0.867
≤32 weeks (%)	22(68.8)	89(79.3)	0.288
Birth weight* (g)	1,415.3±713.6	1,417.4±671.4	0.987
VLBW (< 1,500 g) (%)	22(68.8)	74(61.2)	0.430
ELBW (< 1,000 g) (%)	13(40.6)	46(38.0)	0.787
Apgar score at 1 min.*	3.3±2.6	4.0±2.3	0.161
Apgar score at 5 min.*	5.0±2.9	5.7±2.2	0.054
RDS (%)	21(65.6)	72(59.5)	0.528
BPD (%)	10(41.7)	45(42.1)	0.972
PDA with treatment† (%)	6(25.0)	20(18.7)	0.484
Early sepsis (%)	3(9.4)	8(6.6)	0.590
Abnormal brain sonography‡(%)	6(25.0)	22(20.8)	0.648
Survival during NICU stay (%)	24(75.0)	107(88.4)	0.054
Death within 7 days after birth	8(25.0)	7(5.8)	0.001

* Values are means±SD.

Abbreviations: VLBW, very low birth weight; ELBW, extremely low birth weight; RDS, respiratory distress syndrome; BPD, bronchopulmonary dysplasia; PDA, patent ductus arteriosus; NICU, neonatal intensive care unit

†PDA treated with medication and/or ligation

‡Intraventricular hemorrhage (≥grade 2)±periventricular leukomalacia

The overall survival rate during NICU stay was 75% in twins vs. 88% in singletons, and it was not different statistically between these two groups (*P* = 0.054). However, early death was significantly more frequent in twins (25%) than in singletons (6%) (*P* = 0.001). Among the 15 preterm infants who expired early, 8 were twins and 7 were singleton infants. All of them were less than 1,000 g with a GA below 27 weeks’. In two twin pregnancies, both twins who were 23^+0^ weeks of GA and both twins who were 26^+1^ weeks of GA died. The other 4 infants who expired were the second-born twins from twin pregnancies (Table 3).

**Table 3:** Detailed data of all preterm infants who were admitted to the neonatal intensive care unit and died within 7days from birth.

	Gestational age	Birth weight	Gestational age at cerclage	Days from cerclage to delivery	Hospital days at death
Twins					
1	23^+0^*	610	21^+4^	10	2
2	23^+0^*	550	21^+4^	10	2
3	24^+1^	660	23^+4^	4	5
4	25^+1^	700	20^+6^	30	1
5	26^+1^^†^	760	19^+5^	45	2
6	26^+1^^†^	850	19^+5^	45	2
7	27^+1^	970	22^+2^	34	7
8	27^+2^	940	20^+6^	45	5
Singletons					
1	23^+0^	460	17^+0^	42	2
2	23^+3^	570	22^+0^	10	4
4	24^+0^	760	19^+2^	33	7
5	24^+0^	710	21^+2^	19	3
3	24^+2^	830	22^+6^	9	2
6	26^+0^	780	22^+2^	26	7
7	26^+5^	980	21^+3^	37	1

*Delivered from the same mother

^†^Delivered from the same mother

## Discussion

In this study, we showed that, compared to preterm delivery in singleton pregnancies, EC in twin pregnancies did not increase the preterm delivery rate at ≤32 weeks or ≤28 weeks. Regarding birth weight, EC in twin pregnancies did not increase the delivery rate of either VLBWI or ELBWI compared to those in singleton pregnancies.

A previous study of singleton pregnancies by Lee et al. [12] concluded that EC was the only way to prolong pregnancy for women with advanced cervical dilatation with or without prolapsed membranes in singleton pregnancies. For twins, one study suggested that no treatment, including cervical cerclage, could reduce the risk of preterm birth in asymptomatic twin pregnancies with any indications [3]. Another study of 424 twins with sonographic-indicated cerclage found a similar conclusion that cerclage did not reduce the rate of spontaneous preterm birth when compared to no treatment [26] However, a recent study of 40 dichorionic-diamniotic twin gestations reported that the rate of early preterm birth at <32 weeks of GA was reduced by up to 60% with cervical cerclage [26]. Other studies have also drawn similar conclusions that cervical cerclage including EC in twin pregnancies significantly decreased the rate of spontaneous preterm births [8, 19]. The reason for these conflicting results regarding the efficacy of cerclage in twin pregnancies might be due to different surgical techniques and management protocols used in different institutions [27]. In our study, the rate of preterm birth and the rate of VLBWI or ELBWI in twin pregnancies were not increased compared to the rates in singleton pregnancies.

Recent studies have shown that EC in twin pregnancies improved neonatal outcomes when compared to expectant management [27–29]. Another study also concluded that EC for cervical insufficiency in twin pregnancies and in singleton pregnancies contributed to a good perinatal prognosis [19]. In this study, the incidences of prematurity-related complications were not different in either group, showing that if aggressive antenatal treatment together with adequate postnatal care were provided, the outcomes of twin pregnancies with cervical insufficiency were similar. In 2014, Rebarber et al. [8] found the same results as ours, in that the outcomes of twin and singleton pregnancies were similar if EC was preformed between 14 and 23 weeks of gestation. In addition, they concluded that cerclage should be considered as an option for mothers with twin pregnancies and a dilated cervix during the second trimester.

In our study, the mean GA at the time of EC in twin and singleton pregnancies was 22.3 and 21.8 weeks of gestation, respectively, which is similar to the mean of 22 weeks reported in another study [14]. The mean number of days from cerclage to delivery was 47.9 days in twin pregnancies (range, 4–103 days) and 48.3 in singleton pregnancies (range, 1–141), which are slightly shorter durations than those reported in recent studies by Ehsanipoor et al [14] (54 days) and Namouz et al [11] (56 days). The difference might be due to population differences between the study subjects, i.e., only preterm infants were included in this study vs. all infants including full-term infants were included in other studies.

For neonatologists and obstetricians, preterm birth is a very concerning matter. Even though adequate treatment was provided, the occurrence of some deaths could not be prevented. In addition, preterm twins have higher morbidity and mortality than term twins [25]. A recent systemic review including two prospective cohort studies and seven retrospective cohort studies showed that EC improved pregnancy outcomes compared to no cerclage with a neonatal survival rate of 71% with cerclage vs. 43% with expectant management (relative risk, 1.62; 95% CI 1.19–2.28) [14]. Another study of 43 pregnancies (12 twins and 31 singletons) reported a neonatal survival rate of 83% in twins and 84% in singletons [8]. Another study of 14 patients including twin and singleton pregnancies found a survival rate of 75% [17]. In this study, the overall neonatal survival rate during the NICU stay was 75% in twins and 88% in singletons. Since our study included only preterm infants, this might have contributed to the difference in survival rates when compared to those of other studies. Additionally, although the survival rate seemed to be higher in singleton pregnancies than in twin pregnancies, it was not significantly different (*P* = 0.054), showing that EC was an effective treatment both in twin and singleton pregnancies.

Because we hypothesized that neonatal death at an earlier stage might reflect certain intrauterine conditions, analyzing the mortality rate for infants who expired early (i.e., infants who died within 7 days of hospitalization in the NICU) might provide some information about the efficacy of cerclage and yield different results. Hence, we further evaluated 15 infants who expired early. All the expired infants were less than <1,000 g with GA of ≤27 weeks, and in twin pregnancies, they were the second-born infants in terms of birth order. This suggests that further study to propose concrete indications for intervention is urgently needed to improve the efficacy of EC in twin pregnancies.

The main limitation of our study was its retrospective study design, as it was not a randomized controlled trial (RCT). Considering the ethical aspects of EC, most published data have also been retrospective. Even the only randomized trial by Althuisius et al. [30] contained only 23 recruited patients. However, although there were no subsequuent recruitments that could allow us to view the data as sufficient, their findings did suggest that the time from randomization to delivery was longer in the suture group.

Second, we only studied preterm infants who were admitted to the NICU after at least 23 weeks of GA; hence, if the study subjects were broadened to include some miscarriages or full-term infants, the results might be different. Third, although there might be a different effect between monochorionic-diamniotic twin and dichorionic-diamniotic twin gestations, we did not distinguish any differences between the two. As described in the Methods section, we were only able to acquire descriptions of chorionicity for a small number of cases. Hence, there might be a confounding difference due to complications of monochorionic pregnancies. However, we tried to reduce potential bias by focusing on only preterm infants at the beginning. Lastly, there might have been some differences between the degrees of amniotic membrane protrusion among the women with cervical insufficiency, even within the group of singleton pregnancies or that of twin pregnancies. Hence, not all EC might have been performed under similar conditions, which could confound the results. However, we attempted to use the same diagnostic criteria for EC, regardless of the type of pregnancy.

In addition, the strengths of our study are that it was performed in a single-institution, and infants and their mothers were treated by the same obstetrician (Prof. Lee), who performed all the EC procedures, and the same neonatologist (Prof. Sung) throughout the study period; thus, a uniform management protocol was adhered to throughout the study period. Most of women who underwent EC were followed to delivery at our institution; furthermore, the patient cohort was large.

In conclusion, even though there are currently no concrete treatment guidelines for asymptomatic twin pregnancies with cervical insufficiency, our study might provide some promising results regarding the effectiveness of EC in twins. A recent study by Roman et al [16] proposed a prospective registry of women with a short (<15 mm) and dilated cervix before 24 weeks and found a 49% decreased risk of spontaneous preterm birth. Even though the early mortality rate was high among twins, the overall survival rate during NICU hospitalization was not different between the two groups. Additionally, if proper antenatal care and appropriate EC are performed together with aggressive NICU care, the maternal morbidities and neonatal mortality in twin pregnancies with cervical insufficiency might be the same as those in singleton pregnancies. EC should be performed after considering the indications, contraindications, benefits and risks. A study called the ENCIRCLE trial (Emergency Cerclage in Twin Pregnancies at Imminent Risk of Preterm Birth: an Open-Label Randomized Controlled Trial has already begun in the UK as a randomized control trial, and the data will be available in the near future for twins [31]. Finally, similar to the preterm clinical network database study, a large–scale prospective RCT on this topic is urgently needed [32].
